# Exploring Brazilian Immigrant Mothers’ Beliefs, Attitudes, and Practices Related to Their Preschool-Age Children’s Sleep and Bedtime Routines: A Qualitative Study Conducted in the United States

**DOI:** 10.3390/ijerph15091923

**Published:** 2018-09-04

**Authors:** Ana Cristina Lindsay, Carlos André Moura Arruda, Márcia M. Tavares Machado, Gabriela P. De Andrade, Mary L. Greaney

**Affiliations:** 1 Department of Exercise and Health Sciences, University of Massachusetts Boston, 100 Morrissey Boulevard, Boston, MA 02125, USA; gabriela.deandrad001@umb.edu; 2 Department of Nutrition, Harvard T.H. Chan School of Public Health, Boston, MA 02115, USA; 3 Department of Community Health, Federal University of Ceará, Fortaleza, Ceará 62010-560, Brazil; andrecaninde@yahoo.com.br (C.A.M.A.); marciamachadoufc@gmail.com (M.M.T.M.); 4 Health Studies & Department of Kinesiology, University of Rhode Island, Kingston, RI 02881, USA; mgreaney@uri.edu

**Keywords:** Brazilian, immigrant, parent, sleep, preschool-age children, obesity

## Abstract

In the United States (US), racial/ethnic minority children, low-income children, and children of immigrant families are at increased risk of childhood obesity. Mounting evidence documents that sleep duration and sleep quality are important modifiable factors associated with increased risk of obesity among preschool-aged children. The number of Brazilian immigrants in the US is increasing, yet no existing research, to our knowledge, has examined factors affecting sleep and bedtime routines of children of Brazilian immigrant families. Therefore, the purpose of this qualitative study was to explore Brazilian immigrant mothers’ beliefs, attitudes, and practices related to sleep and bedtime routines among preschool-aged children. Seven focus group discussions (FGDs) were conducted with 37 Brazilian immigrant mothers of preschool-age children living in the US. The audio-recordings of the FGDs were transcribed verbatim in Portuguese without identifiers and analyzed using thematic analyses. Mothers also completed a brief questionnaire assessing socio-demographic and acculturation. Analyses revealed that most mothers were aware of the importance of sleep and sleep duration for their children’s healthy growth and development. Mothers also spoke of children needing consistent bedtime routines. Nevertheless, many mothers reported inconsistent and suboptimal bedtime routines (e.g., lack of predictable and orderly bedtime activities such as bath, reading, etc. and use of electronics in bed). These suboptimal routines appeared to be influenced by day-to-day social contextual and environmental factors that are part of Brazilian immigrant families’ lives such as parents’ work schedule, living with extended family, living in multi-family housing, neighborhood noise, etc. Analyses identified several modifiable parenting practices related to young children’s sleep and bedtime routines (e.g., irregular bedtime, late bedtime, inconsistent bedtime routines, use of electronics in bed, etc.) that can be addressed in parenting- and family-based obesity prevention interventions. Interventions should consider the social context of the home/family (e.g., parents’ work schedules) and the environment (e.g., multi-family housing; neighborhood noise, etc.) faced by Brazilian immigrant families when developing health promotion messages and parenting interventions tailored to this ethnic group.

## 1. Introduction

In the United States (US), racial/ethnic minority children, low-income children, and children of immigrant families are at greater risk of childhood obesity than white, middle- and high-income children [1,2,3,4]. Research suggests that socioeconomic (e.g., family income, lower maternal education, crowded housing, etc.) and environmental factors (e.g., neighborhood characteristics) shape children’s home environment and household routines, and are linked to disparities across most known childhood obesity-related risk factors [5,6,7]. Identifying and addressing early-life risk factors is central to preventing childhood obesity [5,6], which has immediate- and long-term health consequences [1,6]. Reducing childhood obesity among minority, low-income children, and children of immigrant families is crucial due to their increased risk of early childhood obesity in these groups.

Mounting evidence suggests that sleep duration and sleep quality are important modifiable factors associated with increased risk of obesity among preschool-aged children (2–5 years old) [8,9,10,11]. Sleep is essential for healthy cognitive and physical development [9,10,11,12], and lack of sleep, short duration, or poor quality sleep can have adverse effects on children’s physical activity levels, diet quality, as well as the levels of hormones associated with increased risk of obesity [9,11,13,14,15,16,17,18]. Several health organizations including the Health and Medicine Division (HMD) of the National Academies and the American Academy of Pediatrics (AAP) have made recommendations for sleep duration (children 1 to 2 years and 3 to 5 years of age should sleep 11 to 14 h, or 10 to 13 h per 24 h (including naps) on a regular basis, respectively) and sleep hygiene (i.e., consistent bedtimes, wake times, and bedtime routines) to prevent and control obesity in early childhood [19,20].

Several studies have reported a decline in sleep duration over the last decades [9,11,21], with some studies documenting the greatest decline among children under 5, which has been attributed to later bedtimes [10,12,22,23,24,25,26]. Furthermore, research documents that children from low-income households are more likely to have inconsistent bedtime routines than their more advantaged, higher-income counterparts [12,17,27]. Inconsistent bedtimes and no set bedtime routines (i.e., broadly defined as parents engaging their child in the same activities in the same order on a nightly basis prior to lights out) may contribute to disparities in sleep quality and sleep duration that are associated with adverse behavioral, cognitive, and health outcomes including risk of obesity [26,27,28,29,30,31].

Research evidence suggests that the home environment is a central influence on child obesity risk [32,33,34,35] as it is an important social setting where children develop and maintain early health behaviors with immediate and long-term health implications [32]. Parents are critical in establishing a family and home environment that may help their children develop and maintain healthy practices such as sleep and bedtime routines that impact children’s health and weight status [35,36,37,38]. Furthermore, research suggests that there is a relationship between family socio-demographics (e.g., income, race/ethnicity, educational level, etc.) and children’s sleep duration and bedtime routines [39]. For example, children in low-income, immigrant and racial/ethnic minority households are less likely to have regular bedtime routine and screen time limits both of which promote adequate nighttime sleep [40,41]. Lack of parental knowledge regarding healthy sleep habits and the importance of bedtime routines may be associated with negative sleep practices [42,43]. Parenting practices such as setting and enforcing rules (e.g., setting a bedtime), developing family bedtime routines (e.g., bath before bedtime, reading in bed) are associated with healthy sleep patterns for children [44,45]. Additionally, parental screen-time rules (e.g., limiting access to TV and other electronic viewing devices) might positively impact children’s sleep quality and duration [46,47,48].

Social context is influenced by socio-demographic characteristics (e.g., social class, race/ethnicity, language, immigration status) that impact individuals’ and families day-to-day realities [49]. Social contextual factors affect individuals’ and families’ daily practices and behaviors at the intrapersonal (e.g., material circumstances, add another), interpersonal (e.g., family roles, family ties, etc.), organizational (e.g., social services, healthcare, etc.), environmental (e.g., proximity to health centers, neighborhood traffic, etc.), and societal (e.g., social discrimination, isolation, etc.) levels [49].

Brazilians are a fast emerging Latino population subgroup in the US [50]; nevertheless, there is a dearth of research focused on health behaviors and status of Brazilian immigrant children [50,51,52]. Brazilians are a unique group, consisting of people from multiethnic backgrounds (e.g., Africans, Europeans, Asians and Native-Brazilians) [52,53]. Portuguese is the official language spoken by Brazilians, and an important cultural difference between Brazilians and other Latin American population groups [52,53]. The majority of Brazilian immigrants live in the Northeastern US (e.g., Massachusetts (MA), New York (NY), New Jersey (NJ)) [50]. Prior research conducted among immigrant families in the Greater Boston area (MA) showed that 48.2% of Brazilian immigrant children (aged 3–12) were overweight or obese [54].

Understanding parental beliefs, attitudes, and practices related to children’s sleep and bedtime routines, as well as how social contextual factors influence these beliefs, attitudes, and practices, is important for intervention development. This understanding will allow for the identification of modifiable correlates that can be addressed to promote healthy sleep behaviors that may help reduce obesity risk among racial/ethnic minority children who are at increased risk of obesity. Qualitative research methods provide an opportunity to gain an in-depth understanding of Brazilian immigrant mothers’ beliefs, attitudes, and practices related to sleep and bedtime routines of their preschool-age children, which is needed to develop salient parenting intervention to address childhood obesity for this population. Therefore, the purpose of this qualitative study was to explore Brazilian immigrant mothers’ beliefs, attitudes, and practices related to sleep and bedtime routines for their preschool-aged children.

## 2. Materials and Methods

### 2.1. Setting and Sample

This qualitative study was conducted in two cities in MA, US, Somerville and Everett, and is part of a larger ongoing mixed-methods research with Brazilian families living in the Greater Boston area that is exploring parenting styles and parenting practices (e.g., promoting healthy eating, physical activity, and sleep, etc.) related to the risk of childhood obesity [50,51]. Inclusion criteria were: mother with at least one child aged 2–5 years; Brazilian ethnicity, born in Brazil; and living in the US for at least 12 months.

### 2.2. Data Collection

Purposive sampling was used to enroll mothers in the current study. Mothers were recruited through flyers posted at local Brazilian businesses and community-based social and human services agencies, as well as through announcements and events at predominantly Brazilian churches between March and August 2017. Interested women called the telephone number listed on the flyer or spoke to study staff at church events. Participants also were recruited using a snowball technique [55], with mothers enrolled in the study asking their Brazilian friends with preschool-aged children if they would be interested in participating in the study.

This study used focus group discussions (FGDs), as they are valuable research method for working in diverse cultural settings [55,56] as the sharing of ideas and the synergistic effects of the group setting yield rich information and discussion that may not arise in individual interviews [56]. The FGDs were held between April and August 2017 at the churches where recruitment took place. Before each FGD, the moderator explained in Portuguese the study’s purpose, FGD procedures, study confidentiality, and obtained oral and written informed consent from all participants.

One author (ACL), a native Brazilian-Portuguese speaker and qualitative researcher, moderated all FGDs in Portuguese using a semi-structured discussion guide that explored participants’: (1) sleep-related beliefs in general and for young children; (2) attitudes toward bedtime routines; and (3) practices related to their preschool-age children’s bedtime routines. The guide also explored physical activity, and sedentary behaviors including screen time, as well as sources of information used to obtain information about these behaviors with those results reported elsewhere [57,58]. The guide was pilot tested in a FGD with Brazilian immigrant mothers (*n* = 4) and refined before use in the current study.

Before each FGD, the moderator asked participants to think about their preschool-aged children when participating in the discussion. A trained, bilingual (Portuguese and English) research assistant (GDA) took notes during all FGDs, which were audio-recorded and lasted between 60 and 80 min. The moderator and research assistant met for about 15 min at the end of each FGD and reviewed new and recurring themes that were entered into a grid allowed for emerging themes to be tracked and to determine when data saturation had occurred.

Lastly, at the end of each FGD, participants completed a brief, self-administered questionnaire in Portuguese that assessed education, marital status, country of origin, and length of time living in the US. Participants also completed the Short Acculturation Scale for Hispanics (SASH), a 12-item validated scale, to determine acculturation level. The SASH assesses language use, media use, and ethnic social relations [59]. Each item of the SASH is measured on a scale of 1–5 (1 = least acculturated, 5 = fully acculturated), and an acculturation score was computed by averaging across the 12 items.

This study was performed in accordance with the Declaration of Helsinki. This study received ethical approval from the University of Massachusetts—Boston Ethics Board (IRB # 2013060).

### 2.3. Data Analysis

A professional transcriptionist and native Brazilian speaker transcribed all audio-recordings verbatim. The Portuguese transcripts were analyzed using thematic analysis, an iterative process of coding the data in phases to create meaningful patterns [60,61,62], by two experienced qualitative researchers who are native Portuguese speakers (ACL, CAMA). Each researcher read several transcripts numerous times to become familiar with the content and generate initial codes [61,62]. The researchers then manually coded all transcripts independently, but met regularly to discuss coding and to identify and resolve disagreements in coding. The coded text describing similar ideas were grouped and sorted to identify emergent themes and subthemes. Finally, salient text passages were extracted and translated into English to be used as illustrative quotes for the emergent themes [62]. In addition, descriptive statistics and frequencies were calculated for the socio-demographic survey data using Microsoft Excel 2008 (Microsoft, Redmond, WA, USA).

## 3. Results

Seven FGDs (average of five participants per group; range: 4–7) with a total of 37 mothers were conducted before data saturation occurred, with no new themes or subthemes emerging during the final FGD. Of the 37 mothers, seven (approximately 19%) were recruited through the use of snowball sampling technique.

Mothers’ (*n* = 37) ages ranged from 26 to 41 (M = 35.3, SD = 2.8) years [55]. Approximately 92% (*n* = 34) of participants were married and most had two children (*n* = 33, 89%). The majority (72%; *n* = 21) had graduated from high school, was self-employed, and owned a housecleaning business (92%; *n* = 34). Approximately half (51%; *n* = 19) reported a family income of $40,000 or less, which is considered to be a low-middle income. In addition, the majority spoke Portuguese at home (92%, *n* = 34), watched television programs in Portuguese (95%, *n* = 35), and reported that the majority of their friends were Brazilians (87%). Participants were from three regions of Brazil—the Southeast (e.g., Espirito Santo, Sao Paulo, Minas Gerais), the South (e.g., Santa Catarina), the Midwest (e.g., Goias, ato Grosso), with the majority (64.7%; *n* = 22) being from the state of Minas Gerais in the Southeast region. Mothers had lived in the US for an average of 6.7 (SD = 2.84) years, and their mean acculturation score was 1.43 (SD = 0.77), indicating that they identified more closely with Brazilian culture than that of the US.

**Figure 1:**
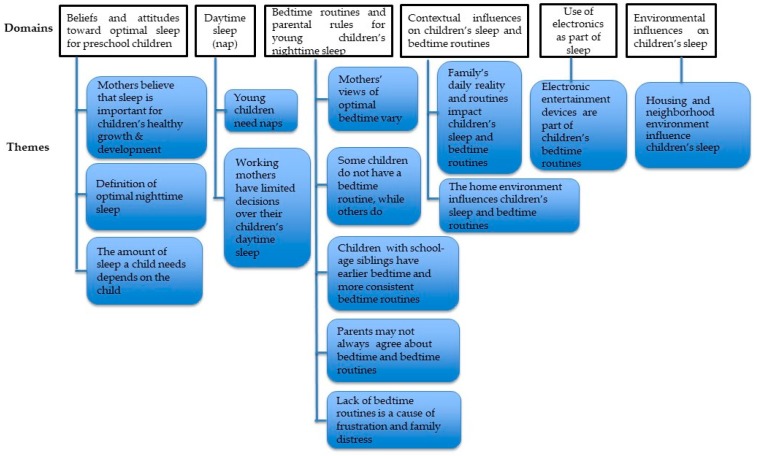
Domains and themes.

Mothers’ beliefs, attitudes, and practices related to their preschool-aged children’s sleep and bedtime routines were categorized into six domains and 14 themes (see Figure 1). The identified themes are exemplified with representative quotes that indicate which FGD participant (M#1–M#37) are being quoted.

### 3.1. Domain 1: Beliefs and Attitudes toward Optimal Sleep for Preschool Children

**Theme 1:** Mothers believe that sleep is important for children’s healthy growth and development.

Across all FGDs, there was consensus about the importance of sleep for the health and wellbeing of young children, with all mothers (*n* = 37) emphasizing the importance of children obtaining adequate sleep for their healthy development and wellbeing.

I think sleep is very important. Children need to sleep well to be healthy and grow and develop. We all need sleep, but I think children need to sleep even more than adults because they are growing and developing…

(M#20; two children—a 8-year-old and a 3-year-old)

**Theme 2:** Definition of optimal nighttime sleep.

Mothers’ defined optimal nighttime sleep for children as sleeping throughout the night without waking up and sleeping for at least 8–9 h without disruption. The majority (*n* = 31) of mothers believed that preschool-age children need a minimum of 8 to 12 h of sleep at night.

*I believe that children need to sleep through the night and not wake up in the middle of the night… that’s okay when they are babies, but when they get to be a little older they should sleep through the night without waking up, that’s very important for them to have solid sleep*.

(M#13; three children—a 10 year-old, a 9-year-old and a 4-year-old)

I don’t know, but I think young children need more than 8 hours of sleep at night. They need like 10 to 11 hours of sleep … I feel when my son doesn’t sleep about 10 hours at night he’s cranky the next day…

(M#17; two sons—a 7-year-old and a 2-year-old)

**Theme 3:** The amount of sleep a child needs depends on the child.

A notable number of mothers (*n* = 20) felt that the amount of sleep an individual child needs varies. Mothers explained that some children may require more sleep based on their energy level and temperament.

*I think children need more sleep at night than adults, maybe like 10 to 11 hours but I think how much sleep a child needs depends a lot on the child. Some children need more sleep than others...like my daughter always slept a lot since she was little. She slept like 12 to 13 hours at night and she needed it. With my son it was different, since he was a baby, he would sleep like 7 to 8 hours and he would be up, full of energy … he took naps, but at night he wouldn’t sleep more than 8 hours straight … and he is fine, very healthy. I think it depends on the child*.

(M#9; two children—a 9-year-old and a 3-year-old)

### 3.2. Domain 2: Daytime Sleep (nap)

**Theme 4:** Young children need naps.

Most mothers (*n* = 29) believed that preschool-aged children need additional sleep time (naps) during the day. Additionally, the majority of these mothers (*n* = 17) felt that younger children (2–3 years old) might need multiple naps for healthy development and wellbeing. Some mothers (*n* = 10) explained that as children get older (4–5 years of age) less daytime sleep is needed.

*…When they [children] are little they need to take 2 to 3 naps during the day, they have a lot of energy but also get very tired and need to rest during the day. As they get older, they need less sleep during the day…*.

(M#30; two children—a 7-year-old and a 4-year-old)

I think all young children need some quiet time during the day even if they don’t fall asleep… they need to take a break…and I also need them to take a break because I need a break myself [laughs]…

(M#18; three children—a 10-year-old, a 7-year-old and a 2-year-old)

Although the majority of mothers (*n* = 29) agreed that preschool-age children need to nap, some mothers (*n* = 12) mentioned that too many (>1–2 naps), or very long (>1–1.5 h) naps result in a later bedtime due to the child not being tired, and children not sleeping through the night without waking up.

*I think young children need additional sleep during the day and should take a nap when they are tired, but I think when they [children] take too many naps or sleep for too long during the day it’s not good, they have a hard time going to bed at night and sleeping for the whole night…at least mine do*.

(M#8; two children—a 5-year-old and a 2-year-old)

**Theme 5:** Working mothers have limited say about their children’s daytime sleep.

Mothers who worked outside the home (majority; *n* = 34) spoke of their work schedule requiring that members of their extended families or paid daycare providers care for their children during the day. These mothers spoke of this reliance resulting in their having limited influence over how much their children slept during the day.

In my case, my two little ones who are not in school and stay with my aunt during the day. I know they take a couple of naps, but I am not the one in charge. Some days I think they sleep more during the day because when I try to get them in bed at around 8:30–9:00 p.m. with my oldest son, they are not tired and it’s hard to get them to want to go to bed…

(M#11; three children—a 8-year-old, a 4-year-old and a 3-year-old)

Several mothers (*n* = 23) spoke of their frustration about this, and of asking relatives (e.g., grandmothers) and daycare providers to limit their children’s naps as they felt napping made their children have a difficult time falling asleep at night.

Mine [children] stay home with my mother during the day and I feel that some days they sleep too much because some nights it’s a battle to get them in bed and they don’t fall asleep until 10:00–11:00 p.m. I have talked to my mother that I don’t want them [children] taking a nap after 4:00 pm…

(M#3; two children—a 4 year-old and a 21-month-old)

*I think children need plenty of sleep and should sleep during the day [nap], but I think when they [children] sleep too much during the day, they have a hard time falling asleep at night and that’s not good for them [children]…I don’t think it’s good [children] to go to bed too late …I have talked with the babysitter a few times, but some days I think they still sleep [nap] too much during the day, but there isn’t much I can do about it*.

(M#7; two children—a 6-year-old and a 3-year-old)

My daughter goes to a daycare and it’s really hard when she takes too many naps during the day because then it’s hard to get her to bed early like 8:30–9:00 p.m. My husband doesn’t mind, but I get really tired and I need to get up early the next day…

(M#21; one child—a 3-year-old)

### 3.3. Domain 3: Bedtime Routines and Parental Rules for Young Children’s Nighttime Sleep

**Theme 6:** Mothers’ views of optimal bedtime vary.

Mothers discussed a variety of approaches to their child’s bedtime. Some mothers (*n* = 16) reported that they were “rigid” and consistent with their children’s onset of nighttime sleep.

At our house I put my son to bed between 8:00 and 9:00 p.m. I’m very strict with his sleep. I have a lot of discipline with his nighttime sleep, and he never wakes up crying to go to school. But every child has a way…

(M#14; one child—a 5-year old)

Conversely, other mothers (*n* = 21) reported that their preschool-aged children had late or inconsistent bedtimes. About half of these mothers (*n* = 11) appeared to accept, and not be concerned about a late bedtime, as their young children did not have to go to school the next day. A few (*n* = 8) mentioned that they expected that their children would have a consistent earlier bedtime once they started school.

My children are still young and not in school, so I am not too worried when they stay up late some nights when they are not tired enough to go to bed. I know that once they get older and start school, they will have more of a routine of going to bed early. That happened to my niece…They [children] get tired and have to wake up early…

(M#23; two children—a 3-year old and a 2-year old)

**Theme 7:** Some children do not have a consistent bedtime routine, while others do.

Many mothers (*n* = 21) reported that they did not have regular bedtime routines for the preschool-aged children.

In our house, we don’t really have a set of routines. I mean, of course the kids brush their teeth before they go to bed, but we don’t really have regular routines. It varies. One thing that my son loves is to watch a cartoon before falling asleep… My kids are still young and can sleep (nap) during the day…

(M#19, two children—a 3-year old and a 2-year old)

In contrast, some mothers (*n* = 16) reported that their children had regular bedtime routines, which often included their child having dinner, taking a bath, brushing teeth, having a bedtime story or praying with child, and the child falling asleep on his/her own with a parent remaining in the room until the child fell asleep.

At our house at around 7 they [children] have dinner, then go upstairs, take a shower, brush teeth and then both [children] go to bed. The older one reads a story by herself, the youngest I have to tell her a make up story. Then we pray and each one turns to her corner to sleep, then I lie down for a few minutes with the little one she falls asleep…

(M#27, two children—a 6-year old and a 3-year old)

In our house, mine [children] are used to having me put them to bed. I put them in bed and pray, I give a little kiss, sometimes my son asks that I read or tell him a short story. So, I tell him a short story and then say now it’s time to go to sleep. They [children] are pretty good. They stay in bed and they fall asleep. The little one sleeps in the same bed with the older one …

(M#6, two children—a 7-year old and a 3-year old)

**Theme 8:** Children with school-age siblings have earlier bedtimes and more consistent bedtime routines.

Mothers with school-aged children (*n* = 16) reported that their school-aged children had early and consistent bedtime routines on school days and that this influenced the bedtime routine of their younger, preschool-age child.

At my house it’s all a routine. We get home, they [children] play some while I get the dinner ready and then they take a bath, get in their pajamas and in bed, we pray and that’s it, the light is out. Since my oldest started school it’s been a pretty nice routine…

(M#26; two children—a 7-year-old and a 3-year-old)

My oldest daughter goes to school, so she needs to go to bed early and read before she falls asleep. So, since my oldest started school, I started putting the younger one in bed about the same time, and now the baby [2-year-old] also goes to bed at the same time. They eat, take a bath, and brush their teeth. The old one reads a book and sometimes I let the little ones watch a cartoon, and they go to bed…

(M#22; three children—a 8-year-old, a 4-yearold, and a 2-year-old)

Overall, mothers who only had preschool-aged and younger (*n* = 21) children, reported less consistent bedtime routines and having family routines that impacted their young children’s sleep and bedtime routine.

We try to have a routine at home and have the children in bed by 9 latest, but it’s not always possible because we have many church activities. Every other week we have a church group meeting at our house, which sometimes lasts until 10 at night. So, the nights that we have church group sometimes they fall asleep on the sofa because there are a lot of people in the house, more noise, they don’t go to bed early, but this is like every fifteen days… my friend tells me it’s more difficult to keep up this schedule when the kids are in school, but mine are not in school yet …

(M#7; two children—a 4-year-old and a 2-year-old)

**Theme 9:** Parents may not agree about bedtime and bedtime routines.

About half of mothers (*n* = 18) reported that their husbands were more tolerant of late bedtime and inconsistent bedtime routines. For some mothers (*n* = 10) this was a source of tension and they spoke of disagreeing with their husbands about their young child’s bedtime and bedtime routines.

My husband and I do not agree about bedtime. He [husband] is fine with the kids staying up late, playing, watching TV with him … my daughter will start school next year and I keep telling my husband we need to get her used to going to bed earlier…

(M#32; two children—a 4-year-old and a 2-year-old)

At my house it’s a constant fight between me and my husband about the children’s bedtime…I’d like the kids to be in bed earlier, but my husband doesn’t care as much. He doesn’t mind the kids being up late. So, we are constantly arguing about it [bedtime]…

(M#21; two children—a 4-year-old and a 3-year-old)

**Theme 10:** Lack of bedtime routine is a cause of frustration and family distress.

Several mothers (*n* = 9) spoke of their young child’s lack of consistent bedtime routines causing frustrations, family distress, and making it difficult for children to fall asleep.

At our house I wish my kids would go to bed earlier. They stay home during the day with my mom and I know they want to stay up and spend some time with me and my husband, but I get tired after a long day of work and it’s always a battle to get the kids to bed, the little one often has these big meltdowns—he gets so tired, but he resists going to bed…he starts crying and we need to try and calm him down to get him to fall asleep. I am always telling my husband, we need to get the kids to bed earlier. I wish we did not have to go through this every night…

(M#17; two children—a 3-year-old and a 2-year-old)

At our house it’s a constant battle with bedtime. My little one [son] does not have a routine, just like my older daughter. If we let, she stays up with everyone [adults] and goes to bed at midnight. They want to stay up with the crowd [adults]. Since last year when my daughter started in Kindergarten she goes to bed earlier …about 9:30… But, my son if we want him to go to bed early, we [adults] have to go to bed early too. He loves to wake up late and since he does not go to school until 11:15 in the morning, I let him sleep until the time he wants…

(M#25; two children—a 6-year old and a 3-year old)

### 3.4. Domain 4: Contextual Influences on Children’s Sleep and Bedtime Routines

**Theme 11:** Family’s daily reality and routines impact children’s sleep and bedtime routines.

Many mothers (*n* = 27) reported that their children’s sleep schedule, amount of sleep, and bedtime routine are influenced by the family’s schedules and needs, such as accommodating family activities (e.g., older siblings afterschool sports, church events, etc.) and parents’ work schedules.

Children kind of get used to the rhythm of the family … like my children have a routine. They need to go to bed early because my husband and I need to wake up early to go to work and I need to take the little one to the babysitter before getting the older one on the bus… so, I think that children are adaptable…

(M#24; two children—a 8-year-old and a 4-year-old)

“I have to work, so my son’s schedule become part of the family schedule. Some days I cannot pick him up from the babysitter until about 7:00–7:30 p.m. So, on those days he [son] eats dinner and takes a bath at the babysitter’s house…but still, by the time we get home, I start dinner, my husband gets home, we eat dinner … it gets late …

(M#11; one child—a 2-year-old)

**Theme 12:** The home environment influences children’s sleep and bedtime routines.

The majority of mothers (*n* = 29) spoke of the home environment (family obligations. parental employment) influencing their children’s sleep and bedtime. Mothers noted that multiple family obligations (e.g., other children, siblings’ afterschool activities, church commitments, etc.) and parents’ working schedules negatively impacted their young children’s bedtime, bedtime routines, and sleep duration.

My oldest son does karate and soccer, and his practices are in the evening because I can only take him after work, so the two little ones need to come along … So, the days my son has karate or soccer we don’t usually get home until about 7:00 p.m. and the kids still need to have dinner…

(M#5; three children—a 9-year-old son, a 4-year-old and a 2-year-old)

On Tuesdays I have church group and the kids come with me, so we don’t get home until 9, so Tuesdays they go to bed later…

(M#7; two children—a 4-year-old and a 2-year-old)

In addition, some mothers (*n* = 16) spoke of the challenge of preparing dinner after work, balancing differing work schedules, and making sure that they adhere to their young children’s bedtime and bedtime routines.

*I usually get home at around 5:45 p.m., after work and picking up the children at babysitter and afterschool. I then have to prepare dinner. Often the kids watch some TV while they have dinner and I clean up. So by the time all is done, it’s close to 8:30. The kids need to take a bath and get in their pajamas and it’s never before 9–9:30 that they are ready for bed… and some days when my husband gets home later they start playing and often are not in bed before 10*.

(M#16; two children—a 4-year-old and a 2-year-old)

### 3.5. Domain 5: Use of Electronics as Part of Bedtime Routines

**Theme 13:** Electronic entertainment devices at bedtime are part of children’s bedtime routines.

About half of the mothers (*n* = 19) spoke of the use of electronic entertainment devices such as tablets, iPads, being part of their young children’s bedtime routine. Many mothers (*n* = 18) reported that they knew that this may negatively impact their children’s sleep quality, but struggled with setting limits or taking away electronics from children.

*There was a time I was letting them [children] use electronics freely, but I saw that it was not good, so I don’t allow it [use of electronic devices] anymore, only the older one has the iPad, but even so, she can only play on Saturday for a little, an hour or two when she has no friends over at the house. But I also do not like taking it [iPad] away from her…I feel bad, but I explain to her that I’m not being bad, I’m trying to do my best because it’s not nice for her to stay the whole time with electronics … it’s hard, these kids are growing up with electronics as part of their daily lives…morning and night*.

(M#11; two children—a 7-year-old and a 4-year-old)

I am trying to monitor my children’s use of electronics, but it is not easy. My little one can only fall asleep watching a movie in his iPad. I tell my husband, this is not healthy, but he thinks as long as he falls sleep that’s fine, but I don’t think so…

(M26; three children—a 7-year old, a 5-year-old and a 2-year old)

Moreover, some mothers (*n* = 16) spoke of being conflicted about their children’s use of electronic devices around bedtime. A few mothers (*n* = 7) reported that using electronics helped their preschool-age children fall asleep and as a result they were part of their children’s bedtime routine. On the other hand, some mothers (*n* = 9) felt that the use of electronics was problematic as it often leads to late bedtime and disrupted their children’s sleep.

My little one [son] only falls asleep watching a cartoon in his iPad. I don’t know what to do. I know it’s not good for him to be on his iPad all the time even when he goes to bed, but it’s the only way he can relax and fall asleep…

(M#5; two children—a 7-year-old and a 2-year-old)

My daughter loves to watch cartoons in bed, but I think it is not good for her. When I let her watch a cartoon, often it leads to a “fight” when I tell her that’s it, you need to fall asleep…but she keeps fighting and ends up falling asleep watching cartoons in bed or going to bed later and upset…

(M#36; one child—a 4-year-old)

### 3.6. Domain 6: Environmental Influences on Children’s Sleep

**Theme 14:** Housing and neighborhood environment influence children’s sleep.

Several mothers (*n* = 8) mentioned that living with extended family (not just children and partner/husband) with varied work schedules disrupted their young children’s sleep and bedtime routines.

My husband’s cousin and his wife are now living with us and they don’t have children and work late, some times when they come home late and start talking with my husband and making noise in the kitchen that can wake my son up …

(M#33; two children—a 4-year-old and a 2-year-old)

More than half of the mothers (*n* = 20) reported that housing arrangements such as crowded homes and living in multi-family housing also impacted their children’s sleep and bedtime routines. In addition, a few mothers (*n* = 6) spoke of noise from neighbors making it difficult for their preschool-age children to sleep.

We live in a multi-family house and the neighbors upstairs are very noisy and stay up late. Some nights it’s really hard for the kids to fall asleep, the TV is loud, or they have the music on … We have tried to tell them, but it is also their house and we try to not have many conflicts …

(M#20; two children—a 6-year-old and a 3-year-old)

Finally, several mothers (*n* = 8) reported neighborhood noise such as living in busy streets with traffic noise affected their children’s sleep.

We live in a very busy street—lots of traffic and noise of cars going up and down the street, so sometimes it’s hard for the kids to fall asleep. A couple of months ago I bought a nightlight with sound for my daughter’s room. I turn it on when she goes to bed to help her relax and fall asleep. My husband and I would like to find another apartment in a quieter street …

(M#4; one child—a 3-year old)

## 4. Discussion

Mounting evidence documents that limited sleep duration and poor quality sleep are associated with increased risk of overweight and obesity among preschool-aged children [63,64,65,66,67,68,69]. Nonetheless, there is a paucity of information on minority immigrant parents’ beliefs, attitudes, and practices related to sleep and bedtime routines of their preschool-age children [66,67,68]. In addition, to our knowledge, none of the extant research has focused on Brazilian immigrant families living in the US. To address this gap, the present qualitative study explored Brazilian immigrant mothers’ beliefs, attitudes, and practices related to their preschool-aged children sleep and bedtime routines.

Study findings revealed that overall mothers participating in this study were aware of the importance of sleep for their children’s healthy growth and development, as well as the importance of nighttime sleep duration and consistent bedtime routines. Nevertheless, most mothers reported late bedtimes and inconsistent and suboptimal bedtime routines (e.g., lack of predictable and orderly bedtime activities such as bath, reading, etc. and use of electronics in bed) that appeared to be influenced by day-to-day social contextual and environmental factors that are part of Brazilian immigrant families participating in the current study. Consistent with previous research, findings from the present study suggest that there is a disconnect between mothers’ awareness of the importance of sleep for their children’s healthy growth and development and their parenting practices [17,28,40,47,66,67,70,71]. These findings suggest that interventions designed for Brazilian immigrant parents must address the link between parental awareness, knowledge, and parenting practices related to sleep and bedtime routines. Moreover, interventions should help parents develop household routines and parenting practices to establish bedtime routines for their preschool-age children. Prior research suggests that improving household routines and parenting practices such as setting and enforcing rules (e.g., setting bedtime) and developing a structured environment including regular family bedtime routines are associated with healthy sleep patterns, reduced sleep problems, and may increase nighttime sleep hours for low-income and racial/ethnic minority children [26,46,47,63,72].

An important finding of the present study was that many mothers reported their young children use electronic devices at nighttime for entertainment and as part of their bedtime routines. This finding is concerning given the growing evidence of the adverse effects of access to and nighttime use of electronic devices have on children’s sleep quality and duration [65,73,74,75,76,77,78,79,80]. These findings indicate the need for interventions to help Brazilian immigrant parents increase their parenting skills to limit the availability of electronic entertainment devices in children’s bedrooms and to discourage children’s use of these devices at nighttime and as part of their bedtime routine. Prior research suggests that screen-time reduction strategies such as parents restricting or monitoring of media use including screen-time have a direct positive impact on children’s sleep quality and duration [75,76,81,82].

The current study revealed that housing (e.g., multi-family, household size, living arrangement etc.) and neighborhood environmental factors (e.g., street noise, traffic, etc.) influence young children’s sleep of participating families. These findings are consistent with prior studies with other minority and low-income population groups [40,71,82], and suggest the importance of taking into account housing and neighborhood environment factors that affect young children’s sleep quality and optimal sleep duration. Therefore, interventions aimed at promoting healthy sleep habits and routines among Brazilian children living should consider housing and neighborhood factors such as crowded housing and urban neighborhoods environmental factors (e.g., street noise) to inform the development of interventions for this population.

Finally, mothers participating in the current study had lived in the US for an average of 6.7 (SD = 2.84) years, and had a mean acculturation score of 1.43 (SD = 0.77), indicating that they identified more closely with the Brazilian culture than with that of the US. Previous studies with other immigrant Latino populations have found that sleep quality and duration is associated with acculturation levels [83]. Therefore, additional studies with Brazilian immigrant families are needed to explore the relationship between parents’ acculturation levels and parental beliefs and parenting practices related to sleep of their preschool-aged children. This information will be important for design of interventions that are culturally relevant to immigrant Brazilian parents.

This study has several limitations. Study findings are based on a purposeful sample of low-income, Brazilian-born immigrant mothers in two MA, US communities, which limits generalizability. There is also the possibility of selection bias as mothers with a heightened interest in or awareness of the importance of child health behaviors may have been more likely to participate. Moreover, the use of snowball sampling to recruit participants might have resulted in the recruitment of study participants who share similar beliefs, attitudes, and practices related to preschool-age children’s sleep and bedtime routines. Thus, further research is needed to determine if study’s findings are generalizable to a broader group of Brazilian immigrants living in the US. Future research can build on the present study by exploring the beliefs, attitudes, and practices related to sleep, bedtime and bedtime routines of a broader group of Brazilian immigrant parents, as well that of immigrant parents from other minority groups and communities across the US. Nevertheless, the use of qualitative methods provided deep insight into Brazilian immigrant mothers’ personal beliefs, attitudes and practices about their young children’s sleep and bedtime routines.

## 5. Conclusions

This is the first study, to our knowledge, to explore maternal beliefs, attitudes, and practices related to sleep and bedtime routines among preschool-age children of Brazilian immigrant families living in the US. Study findings revealed modifiable parenting practices (e.g., late bedtimes. inconsistent bedtime, lack of bedtime routines, use of electronics in bed, etc.) related to young children’s sleep and bedtime routines that can be addressed by parenting- and family-based health promotion interventions. Furthermore, findings suggest the influence of the home environment and social contextual factors on children’s sleep and bedtime routines. Therefore, interventions developed to promote healthy sleep habits and routines for Brazilian immigrant children in the US should incorporate an understanding of the social context of the family (e.g., parents’ work schedule; siblings, etc.) and the home environment (e.g., number of adults in the household; housing, etc.). Consideration of these factors will be important for the development of successful interventions attuned with the day-to-day realities of low-income, immigrant Brazilian families.

## Abbreviations

AAP	American Academy of Pediatrics
BMI	Body Mass Index
EDE	Electronic Device for Entertainment
FGD	Focus Group Discussion
IOM	Institute of Medicine
MA	Massachusetts
SASH	Short Acculturation Scale for Hispanics
US	United States
